# The activity of French Research Ethics Committees and characteristics of biomedical research protocols involving humans: a retrospective cohort study

**DOI:** 10.1186/1472-6939-6-9

**Published:** 2005-10-17

**Authors:** Evelyne Decullier, Véronique Lhéritier, François Chapuis

**Affiliations:** 1 UF de méthodologie en recherche clinique, Département d'Information Médicale des Hospices Civils de Lyon, Lyon, France; 2 Hôpital Edouard Herriot, Lyon, France; 3 French National Conference of Research Ethics Committees, Lyon, France

## Abstract

**Background:**

Clinical trials throughout the world must be evaluated by research ethics committees. No one has yet attempted to clearly quantify at the national level the activity of ethics committees and describe the characteristics of the protocols submitted. The objectives of this study were to describe 1) the workload and the activity of Research Ethics Committees in France, and 2) the characteristics of protocols approved on a nation-wide basis.

**Methods:**

Retrospective cohort of 976 protocols approved by a representative sample of 25/48 of French Research Ethics Committees in 1994. Protocols characteristics (design, study size, investigator), number of revisions requested by the ethics committee before approval, time to approval and number of amendments after approval were collected for each protocol by trained research assistant using the committee's files and archives.

**Results:**

Thirty-one percent of protocols were approved with no modifications requested in 16 days (95% CI: 14–17). The number of revisions requested by the committee, and amendments submitted by the investigator was on average respectively 39 (95% CI: 25–53) and 37 (95% CI: 27–46), per committee and per year. When revisions were requested, the main reasons were related to information to the patient (28%) and consent modalities (18%). Drugs were the object of research in 68% of the protocols examined. The majority of the research was national (80%) with a predominance of single-centre studies. Workload per protocol has been estimated at twelve and half hours on average for administrative support and at eleven and half hours for expertise.

**Conclusion:**

The estimated workload justifies specific and independent administrative and financial support for Research Ethics Committees.

## Background

Clinical trials in biomedical research throughout the world must be evaluated by research ethics committees. No one has yet attempted to clearly quantify the activity of ethics committees and describe the characteristics of the protocols submitted, and only five studies[1-4] have dealt with this subject, two in England and the others in the USA, Australia and Spain. However, they each focused on only one or two research ethics committees, and no knowledge is available on a national or even a regional level. In addition, four of these studies[2-4] concentrated on the fate of protocols; some characteristics were gathered but they were presented only for protocols whose investigator was not lost to follow-up. None of these studies evaluated research ethics committees' workload, but one study[1] described the activity in terms of number of meetings, approvals and reasons of queries.

France was one of the first country to affirm these principles through the Huriet-Sérusclat Act of 1988 [5], which launched administrative and financially independent research ethics committees (REC) in 1991, called CCPPRBs (committees for the protection of human beings involved in biomedical research). Each protocol involving human beings in France has to be assessed by one of these committees.

A European harmonization was needed since a long time and was decided in 2001 [6]. All European governments were asked to change national laws according to this directive. Research ethics committees will be created where they do not exist currently and it is of upmost importance to anticipate activity, workload, financial and administrative support needed by these committees.

We describe one year of activity in a sample comprising half of the French RECs and the full characteristics of the protocols approved in these RECs during this year.

## Methods

All biomedical research protocols approved by a representative random sample of every other French RECs the participating committees between 01 January 1994 and 31 December 1994 were included. This year was chosen in order to allow studies to be completed and so to collect data on all amendments, information and serious adverse effects.

### Definitions

The definitions used in this study are drawn from French law on the subject (table 1).

**Table 1:** Definitions

Area covered by the law	Every trial or experimentation involving human beings based on a procedure formalized in a protocol in order to develop biological or medical knowledge.
CCPPRB	"Comités Consultatifs de Protection des Personnes se Prêtant à la Recherche Biomédicale", i.e., committees for the protection of human beings involved in biomedical research.
Composition of REC	The committees gather 12 members and 12 replacements. The REC is made up of four persons competent in biomedical research (at least three physicians), a general practitioner, two pharmacists (at least one working in a hospital, a nurse, one person qualified in ethics, one social worker, one person qualified in psychology and one person qualified in the legal aspects of research.
Studies without direct individual benefit	None of the participants could expect any individual and immediate benefit, e.g. research in physiology or phase I studies are typically considered as studies with no therapeutic benefit for subjects.
Studies with direct individual benefit	Patients can potentially expect a therapeutic benefit from the research.
Decision	RECs have four possibilities in their decisions: rejecting the protocol, accepting the protocol without any modifications, accepting the protocol with minor modifications or asking for revisions (major modifications) before approval.
Time to decision	RECs must give their first answer within 5 weeks after the protocol application.
Revision	Mandatory modification requested by the REC before protocol approval.
Amendment	Modifications proposed by the investigator to the committee after approval
Information	Investigators can send information concerning their study (state of inclusions, article, etc.). This information does not lead to a decision by the REC.

### Data collection

Ethics committee activity and protocol characteristics were gathered into four thematic groups. The first two comprised committee activities: (i) the process of approval (modifications requested before approval, approval date, etc.), (ii) the modifications requested after approval and the information transmitted. The other two comprised protocol characteristics: (iii) the legal and administrative characteristics of protocols (investigator, sponsor, etc.) and (iv) the proposed scientific characteristics (duration, sample size, etc.). Research assistants in charge of data collection were trained in order to ensure homogeneous results. The questionnaires were completed using the committee's archives, and were then sent to the coordinating centre. An identification number was given to each protocol in order to ensure anonymity of the investigator.

### Assessment of activity and expertise workload

#### Activity

Each formal step was studied, i.e., the number of protocols approved, revisions requested by the ethics committee and amendments submitted by the investigator were studied. The number of revisions, amendments and total exchanges (defined as the sum of revisions and amendments) were calculated per approved protocol.

#### Administrative and expertise load

At the REC level, each protocol is registered and assessed for approval. A list of the different tasks was established by the Lyon B REC members and administrative staff and submitted to a national panel of REC members (table 2). The time required to accomplish each task was estimated based on individual experience and checked on site. For this study, the estimated times devoted to administrative work and expert assessment were then accepted by consensus among administrative staff and REC members respectively. The workload is estimated from a protocol perspective, i.e. each protocol is valued. We did not assess neither fix activities, such as or training of the members or structure management (accounting, legal, insurances, structure organisation,...), nor the committee meetings.

**Table 2:** Development of REC activities

	**Main tasks**	**administrative time (h)**	**expert time (h)**
New file	Receipt	0.25	0.25
	Computer registration	0.25	0,25
	Content and conformity checking	1	1
	Acknowledgement of receipt	0.5	-
	Sending to the experts	0.5	-
	Inscription of committee's meeting agenda	0.5	-
	Sending the reply ready for posting	1	-
	Filing	0.5	0.5
	Valuation+writing and sending valuer's report	-	4
	
	**TOTAL NEW FILE**	**4.5**	**6**

Revision	Request for information to the investigator	1	-
	Disfiling	0.5	0.5
	Reply receipt	0.5	0.25
	Sending to the experts	0.5	-
	Inscription of committee's meeting agenda	0.5	-
	Sending the reply ready for posting	1	-
	Filing	0.5	0.5
	Valuation+writing and sending valuer's report	-	2
	
	**TOTAL REVISION**	**4.5**	**3.25**

Amendment	Receipt	0.5	0.5
	Disfiling	0.5	0.5
	Sending to the experts	0.5	-
	Inscription of committee's meeting agenda	0.5	-
	Writing and sending the reply	1	-
	Filing	0.5	0.5
	Valuation+writing and sending valuer's report	-	1

	**TOTAL AMENDMENT**	**3.5**	**2.5**

-: not applicable

### Statistics

As this was mainly a descriptive study, we presented frequency distributions calculated with SAS software^®^. A Kaplan-Meier survival analysis [7] was performed with SPSS software^® ^to establish probability of approval curves based on cumulative hazard function in order to study the time between the submission of the protocol and final approval (in days). A log-rank test [7] was then computed to compare protocols with direct approval, protocols with minor modifications and protocols with revisions.

A multiple correspondence analysis [8] was also performed on protocol characteristics. To determine the number of axes we used the scree test [9] and the total inertia was computed with Benzecri formulae [10]. To explain the meaning of each axis, modalities of variables were cumulated until 80% of inertia was explained. The individual coordinates were then used to obtain a hierarchical clustering with the Ward minimum variance method [11]. The number of clusters was chosen using the cubic clustering criterion [12].

To compare number of revisions, amendments and time per protocol between the clusters an ANOVA was performed [13].

## Results

A total of 25/48 RECs throughout France participated in this national study. There were 1143 declared protocols approved by these committees during the year 1994. One hundred sixty-seven had to be excluded because the inclusion criteria were not fulfilled (Table 3). Thus 976 protocols were eligible (mean per committee, 39; median, 37; range, 17–81).

**Table 3:** Reasons for non-inclusion (167 protocols out of 1143)

	*n*	%
Approved in 1995	82	49
Withdrawn before approval	48	29
Rejected	16	9
Not within the scope of the law	12	7
Missing file	6	4
Approved in 1993	3	2

### REC activity

#### Approval

Only 31% of protocols were approved with no request for modifications. Minor or major modifications were requested for the remaining protocols.

#### Time from submission of application to approval

**Figure 1:**
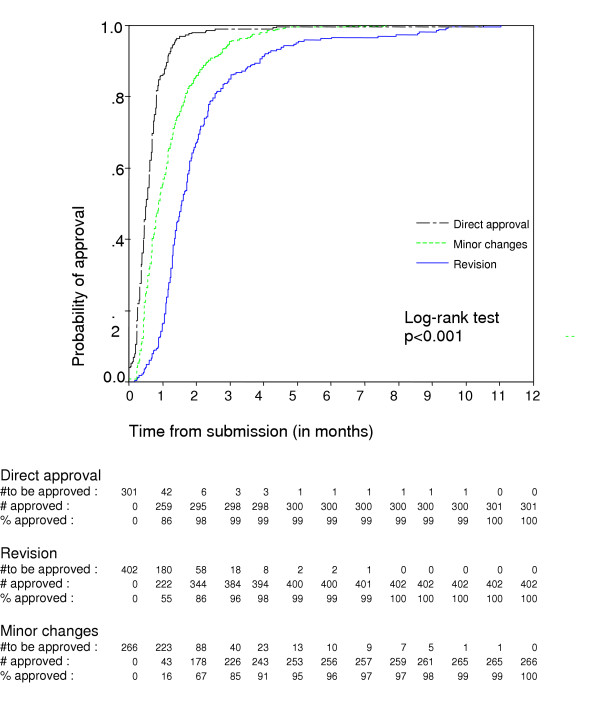
Probability of approval from time of submission, according to the initial decision of Research Ethics Committees.

Because of missing registration and/or approval dates, seven protocols were dropped from the study, and the analysis was performed on 969 studies. The median time to approval for protocols with direct approval, with minor changes requested, and request for major changes (revisions) was, respectively, 16 days (95% confidence interval: 14–17), 27 days (24–30) and 48 days (43–52). The comparison between the three groups resulted in a significant log-rank test (figure 1).

#### Number of modifications studied

On average, the number of revisions required was 39 per committee and per year (median, 30; range, 4–132, 95% CI, 25–53) and the number of amendments was 37 (median, 28; range, 3–91, 95% CI, 27–46). Thus the average number of exchanges (revisions and amendments) was 76 (median, 60; range, 19–212, 95% CI, 55–96) per year and per committee, ranging from 13 exchanges for phase I studies on drugs, through 19 for studies not investigating drugs and to 45 for studies on drugs other than phase 1.

#### Revisions (major changes)

Revisions were requested for 555 (57%) protocols, but only 181 (19%) required a second revision or more. A revision could contain one or more points to be modified by the investigator. In total 1438 points for modification were cited (Table 4). Per protocol, the average number of reasons per revision was 2.01 (the same reasons could have required more than one revision). On average, one reason (or less) was mentioned in 45% of revisions, between one and two reasons in 25%, between two and three 15%, and more than three reasons in 15% (data not shown).

**Table 4:** 1211 main reasons for revision before approval (out of a total of 1438)

	*n*	%
Patient information	399	27.7
Consent modalities	257	17.8
Inclusion criteria	116	8.1
Scientific prerequisite	105	7.3
Legal requirements	91	6.3
Sample size	71	4.9
Insurance	49	3.4
Information on treatments and exams	48	3.3
Study objectives	44	3.1
Information on methodology and statistics	31	2.2

The main reasons for revisions were information to the patient (28% of responses), consent modalities (18%), inclusion criteria (8%), scientific factors (7%) and legal and administrative requirements (6%). This global ranking of reasons remained the same when considering the sub-group of protocols with only one revision and the sub-group of protocols with two revisions.

The ranking of reasons for protocols with three or more revisions showed that legal and administrative points, which were quite easy to solve, became less important, falling from the 4^th ^position (for protocols with one or two revisions) to the 9^th ^position. However, requests for more specific methodological and statistical information, which were more complex to solve, were more frequently cited, rising from the 11^th ^rank (for protocols with one or two revisions) to 7^th ^rank.

#### Amendments (after approval)

Over half of the investigators submitted no amendments (57%), although few protocols were not implemented and therefore could not have led to amendments. For the 416 protocols requiring at least one amendment, 875 reasons were mentioned. An average of 1.2 reasons per amendment was cited. A single reason was cited in 52% of cases.

The main reasons for which protocols had to be modified after approval were a change in the number of subjects to be included (18%), a modification of inclusion criteria, a modification of the timetable and changes in examinations and treatments (Table 5). Regarding the legal and administrative points cited, 43% concerned the update of the list of investigators (data not shown). The number of amendments submitted did not modify the ranking of these reasons.

**Table 5:** 657 main reasons for amendment after approval (out of a total of 875)

	n	%
Sample size	156	17.8
Inclusion criteria	143	16.3
Timetable	132	15.1
Treatment or exam modifications	89	10.2
Patient information	76	8.7
Legal and administrative modifications	61	7.0

#### Additional information

As information on the progress of research is not mandatory, 81% of investigators did not send any further information regarding their study. When they did it was mainly to warn of the end (premature or normal) of the study (30%), to declare adverse side effects (25%) and to inform the committees of the intermediate or final study results (18%).

### RECs' administrative and expertise workload

The administrative staff panel estimated that administrative tasks (registration, postal service, copying, classification, letters, etc.) required 4.5 hours per initial protocol recording, 4.5 hours per revision and 3.5 hours per amendment (table 2). The experts panel estimated that the expertise process required 6 hours per protocol, 3.25 hours per revision and 2.5 hours per amendment. This was confirmed during the test on-site.

These figures show, per year and per month respectively, an estimated administrative workload of 480.50/40 hours and an estimated expertise workload of 453.25/38 hours per REC (based on observed mean annual activity per committee: 39 protocols, 39 revisions and 37 amendments on average per REC and per year).

### Protocol characteristics

Table 6 summarizes the main legal and administrative characteristics of protocols and Table 7 the main scientific and technical characteristics.

**Table 6:** Administrative characteristics of approved protocols

	*n*	%
***Main investigator's status***		
Professor	567	58.1
Assistant	255	26.1
Other	154	15.8

***Sponsor***		
Pharmaceutical firm	628	64.3
Tertiary teaching hospital	161	16.5
Industry	45	4.6
Other public organisation	102	10.4
Other	40	4.1

***Place of research***		
Phase I specialized unit	162	16.6
Tertiary teaching hospital	543	55.6
Tertiary teaching hospital + private hospital	121	12.4
Tertiary teaching hospital + phase I specialized unit	18	1.8
Other	132	13.5

**Table 7:** Technical characteristics of approved protocols

	***n***	**%**
***Topic***		
Drug	667	68.3
Phase I	163	24.4
Phase II	173	25.9
Phase III	226	33.9
Phase IV	105	15.7
Cosmetics and nutrition	50	5.1
Physiology	65	6.7
Medical equipment & prothesis	64	6.6
Surgical and/or diagnostic act	52	5.3
Other	78	8.0

***Design***		
Descriptive, analytic	150	15.4
Experimental, non-randomized	319	32.7
Experimental, randomized	507	51.9

***Scope***		
National-monocentric	463	47.4
National-multicentric	311	31.9
International	161	16.5
Not available	41	4.2

***Expected sample size***		
Fewer than 21 patients	293	30.0
21 to 50 patients	251	25.7
51 to 150 patients	192	19.7
More than 150 patients	228	23.4
Not available	12	1.2

***Expected duration***		
Less than 2 months	115	11.8
2 to 6 months	159	16.3
6 to 18 months	286	29.3
More than 18 months	163	16.7
Not available	253	25.9

#### Legal and administrative characteristics

Sponsors were mainly pharmaceutical firms (64%). The research setting was a tertiary teaching hospital in 55% of cases; another 15% of protocols were conducted simultaneously in a tertiary teaching hospital and another type of institution. The item "other" included other combinations of these settings and also private offices, etc.

#### Technical characteristics

Drug evaluation was the object of research in 68% of cases. For the research topic, the item "other" was cited in 8% of cases, usually for studies on genetics or vaccines, i.e., other types of clinical trials. It was also pointed out that participating RECs encountered problems in reporting expected duration since 26% of the answers on questionnaires neglected to mention this information.

Reasons for revisions varied according to investigators' status (p < 0.0001; revisions on scientific prerequisite were more frequent when investigators were neither professor, nor assistant) and to place of research (p = 0.005; revisions on inclusion criteria were more frequent when research was conducted in phase I units) and reasons for amendments according to sponsor (p = 0.01; amendements on sample size were more frequent when the sponsor was a public hospital), place of research (p = 0.03; amendments on treatment was more frequent for research conducted in phase I units), study phase (p = 0.04; amendements concerned more often treatment for phase I studies), study scope (p = 0.002; sample size was more often modified when the study was national and multicentre) and study duration (p = 0.001; amendments were more often about treatment when study duration was less than 2 months).

#### Multiple correspondence analysis

**Figure 2:**
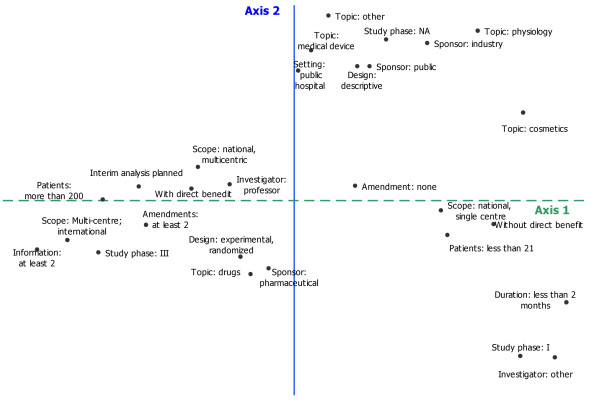
Graphical representation of multiple correspondence analysis on protocols characteristics*. *graphical representation of the modalities which contributes the most to the construction of axes 1 and 2.

the scree test resulted in the selection of two axes explaining 40% of the inertia. The modalities most contributing to the construction of each axis were represented on figure 2. The first axis (dashed line) showed that study size differentiated protocols the most. On one side were phase I studies, protocols studying nutrition, cosmetics, protocols on physiology, without direct benefit to the patient, of short duration, single-centre studies, and those with fewer than 20 patients expected (small studies). On the other side were phase III studies, those with direct benefit to the patient, more than 200 patients to be included, national multicentre or international studies, and those with a planned intermediate analysis (large studies).

The second axis separated descriptive studies sponsored by the public sector from experimental studies on drugs, mostly sponsored by private pharmaceutical firms.

#### Hierarchical clustering using Ward's minimum variance

This analysis yielded four clusters explaining 75% of the variance. The first cluster included protocols on drug testing (98% in this cluster *vs*. 68% in the global population), with direct individual benefit, and phase II and phase IV studies (respectively, 35% and 22% *vs*. 18% and 11%). Cluster 2 grouped phase I drugs trials (89% *vs*. 17%), without direct individual benefit (97% vs. 34%), in a single centre (99% *vs*. 47%), lasting less than 2 months and sponsored by pharmaceutical firms. Cluster 3 also concerned drugs, but protocols were phase III studies (64% *vs*. 23%), with direct individual benefit, multicentre and international (42% *vs*. 16%) and with a randomized design (87% *vs*. 52%). Finally, cluster 4 grouped protocols not evaluating drugs (89% *vs*. 32%), the studies were descriptive (32% *vs*. 15%) and the sponsor was in the public sector (58% *vs*. 27%).

### Activity and workload in relation with protocol characteristics

We have linked the different clusters to the number of revisions and amendments (table 8). The mean number of revisions is homogeneous through clusters with a minimum of 0.94 for cluster I and a maximum of 1.08 for cluster III. Number of amendments varied more: from 0.46 for cluster II to 2.14 for cluster III.

**Table 8:** Tendendy in characteristics of 976 protocols according to clusters defined by a hierarchical analysis

	CLUSTER I	CLUSTER II	CLUSTER III	CLUSTER IV	
N	280	144	209	343	
Main characteristics	-drug testing -phase II -pharmaceutical sponsor -with direct benefit -phase IV	-phase I -without direct benefit -investigator: other -single centre -less than 2 months	-international scope -phase III -more than 200 patients -drug testing -with direct benefit	-not drugs -public setting -descriptive design -public sponsor -industrial sponsor	

PER PROTOCOL					ANOVA p-value
Mean number of revisions	0.94	1.05	1.08	0.98	0.70
Mean number of amendments	0.78	0.46	2.14	0.53	<0.0001
Estimated average time (initial evalution, revision, amendment)	22.5	21.4	31.7	21.3	<0.0001

The mean time spent per protocol (workload) was estimated in each cluster, cluster III protocols are the most time-consuming.

Time spent by each cluster was obtained by multiplying number of protocols by mean time per protocol. At a national level 31% of committees' time is spent for cluster IV protocols, 28% for cluster III, 27% for cluster I and 13% for cluster II.

## Discussion

On average, each REC studied 39 protocols, 39 revisions and 37 amendments per year, representing an annual workload of 934 hours (480.50 hours for administrative tasks and 453.25 for expertise tasks, on a monthly basis respectively 40 and 38 hours) including neither committee meetings nor training of the members since there is too much variability across committees. Most protocols evaluated drugs (68%), were experimental (85%) and were monocentric (47%).

The main advantage of our study is to have collected exhaustive information for half of French RECs over a full year (whether or not the principal investigator was lost to follow-up). Moreover, homogeneous data collection was guaranteed by the initial training followed by research assistants. The choice of the year 1994 was based on initial studies conducted by the French Ministry of Health [14-16] showing that on average six years were needed to reach publication, and that some studies were still ongoing eight years later. This choice enabled us to gather all the information during the whole protocol's duration. To our knowledge our study is the first to have evaluated the workload and the activity of RECs for each protocol through the collection of the number of revisions, amendments and additional information. The combination between 1994's activity data and 2004's workload assessment is justified since committees' missions have remained the same and there is no reason why the registration or the expert evaluation should take more or less time in 1994 compared to 2004. A 2001 French Senate report [17] showed similarities across years in the number of protocols evaluated by French RECs (total number of protocol, type of research,...). Moreover, this would make more sense to use the current procedure and workload (2004) rather than the old ones (1994). To our mind, this evaluation was closest to the reality, since a retrospective cohort was mandatory to evaluate characteristics and fate of biomedical protocols, whereas retrospective data collection was not possible to assess workload.

Moreover the French law is broader than EC 2001/20 European Directive [6], since it aims to protect human beings in all biomedical research (drugs, biomedical devices, physiology, vaccines,...), and therefore the protocols included in our study are not only on drugs evaluation.

The major difficulties encountered concerned definitions. Although the law clearly defines what are direct approvals and revisions, some RECs evocated direct approval with revision and postponed approval without revision. However this issue is not related to our study methods but to a difficulty in understanding and putting into effect the French law. Despite their training, research assistants had difficulties to retrieve information on study design and to categorise it. Some data were often missing in the protocols, such as the expected duration.

The four articles previously published collected information on only one or two ethics committees[1-4]. These studies did not describe the general situation at a country level and they provided information only when the investigator was not lost for follow-up. Only one study gave information on RECs' activity [1], namely some information on the number of new applications, correspondence, decisions, and the number of meetings needed to obtain approval were given.

One of our major results is that 46% of revisions concerned patient information and consent.

The above article showed that when studies were conditionally approved, deferred or rejected, most queries also concerned the patient information leaflet (85%). But only protocols with five or more centres were assessed; consequently these protocols were not representative of all biomedical research on human beings.

A French report [17] also gave such information, but the aim was more to point out problems of putting the law into effect in France.

Almost all protocols anticipate recruitment in terms of sample size rather than in terms of study duration. We think that the first point shows compliance with good clinical practices [18], and the latter expresses the need for a better training of prime investigators for clinical research study management and anticipation.

The workload theme in terms of numbers of protocols, number of revisions and number of amendments is very relevant since French RECs' members work for free during or after their workday, and at least administrative and financial support of the REC structure is obviously needed in order to guarantee independence in each country. The results of our study are fully supported by the recommendations of the Ad Hoc advisory group on the operation of NHS Research Ethics Committees [19], asking for independent opinion, managed operating system, time recognition and protection, as well as finances.

Across countries, RECs may not have the same structure (frequency of meetings, number of people involved in the committee), the same remit (only studies with intervention on human or also studies on physiology, for instance) and the same functioning since the number of protocols reported in the above article was very heterogeneous in the different countries. When looked at in terms of protocols approved per year and per committee, clear differences appeared: 110 protocols approved in the multicentre REC of London [1], 158 protocols in Barcelona [4], 180 protocols in Oxford [2], 94 protocols in Sydney [3] and on average 39 protocols in France.

It would be of great importance to launch a similar study in more recent years and in other settings at country level to see if things have changed, and if so in which way. Studies in different European countries will allow to collect information which would be very useful to ease harmonisation. It would also be interesting to launch a prospective recording of all tasks and time needed on a random sample of committees.

## Conclusion

Up to now, one study described the activity of one REC specialised in multicentre trials and three other studies were carried out on the fate of protocols approved by one committee but nothing was known at a nation level.

Our study showed that 976 protocols were approved in one year by half of the French RECs and that median approval time ranges from 16 days (if no modification) to 48 days. Moreover, most protocols were carried out in France only (79%) for drugs evaluation (68%)

Revisions before approval relates first and foremost to patient information and consent modalities (46%). For a protocol evaluation (first evaluation, revisions and amendments), scientific and administrative workload varied on average from 21.3 hours up to 31.7 hours according to protocols' characteristics.

## Competing interests

The author(s) declare that they have no competing interest.

## Authors' contributions

ED coordinated the study, managed the data, performed the statistical analysis and drafted the manuscript.

VL participated in the design of the study, coordinated the study and managed the data.

FC designed, submitted and coordinated the study, interpreted data, and helped to draft the manuscript.

## Pre-publication history

The pre-publication history for this paper can be accessed here:


